# Solitary pleural metastasis from renal cell carcinoma: a case of successful resection

**DOI:** 10.1186/s40792-015-0039-z

**Published:** 2015-04-23

**Authors:** Masafumi Kataoka, Yuji Yata, Sohichiro Nose, Koichiro Yasuda, Toshinori Ohara

**Affiliations:** Department of Surgery, Okayama Saiseikai General Hospital, 1-17-18 Ifukucho, Kita-ku, Okayama City, 700-8511 Japan; Department of Pathology, Okayama Saiseikai General Hospital, 1-17-18 Ifukucho, Kita-ku, Okayama City, 700-8511 Japan

## Abstract

While renal cell carcinoma frequently metastasizes to the lung, solitary pleural metastasis without lung involvement is extremely rare. A 69-year-old man was admitted to our hospital with a solitary pleural metastasis 6 years after surgery for renal cell carcinoma. Needle biopsy was performed, and the tumor was diagnosed as a metastasis of renal cell carcinoma. The pleural tumor was surgically resected. The patient received interferon-α as postoperative therapy. He has been alive for 9 years without recurrence. Only 11 cases of solitary pleural metastasis have been reported thus far, and of these, 7 involved a large amount of pleural effusion resulting in a poor prognosis. This is the first reported case of solitary pleural metastasis from renal cell carcinoma, which was curatively resected, as indicated by long-term survival.

## Background

Renal cell carcinoma (RCC) accounts for approximately 2% of all cancers, and nearly 50% of all RCC patients will eventually present with metastatic disease, requiring individualized treatment decision. Patients with untreated metastatic disease have a 5-year survival rate of 0% to 18% [1]. A notable feature of RCC is its tendency to result in lung metastases, which were found in 55% of cases in an autopsy-based study [2]. Pleural metastasis is comparatively rarer than lung metastasis. Most pleural metastases are associated with metastatic lesions of the lung. Solitary pleural metastasis without lung metastasis is extremely rare. Herein, we report successful treatment by resection in a patient with a solitary pleural metastasis evidently derived from a previously removed RCC.

## Case presentation

**Figure 1:**
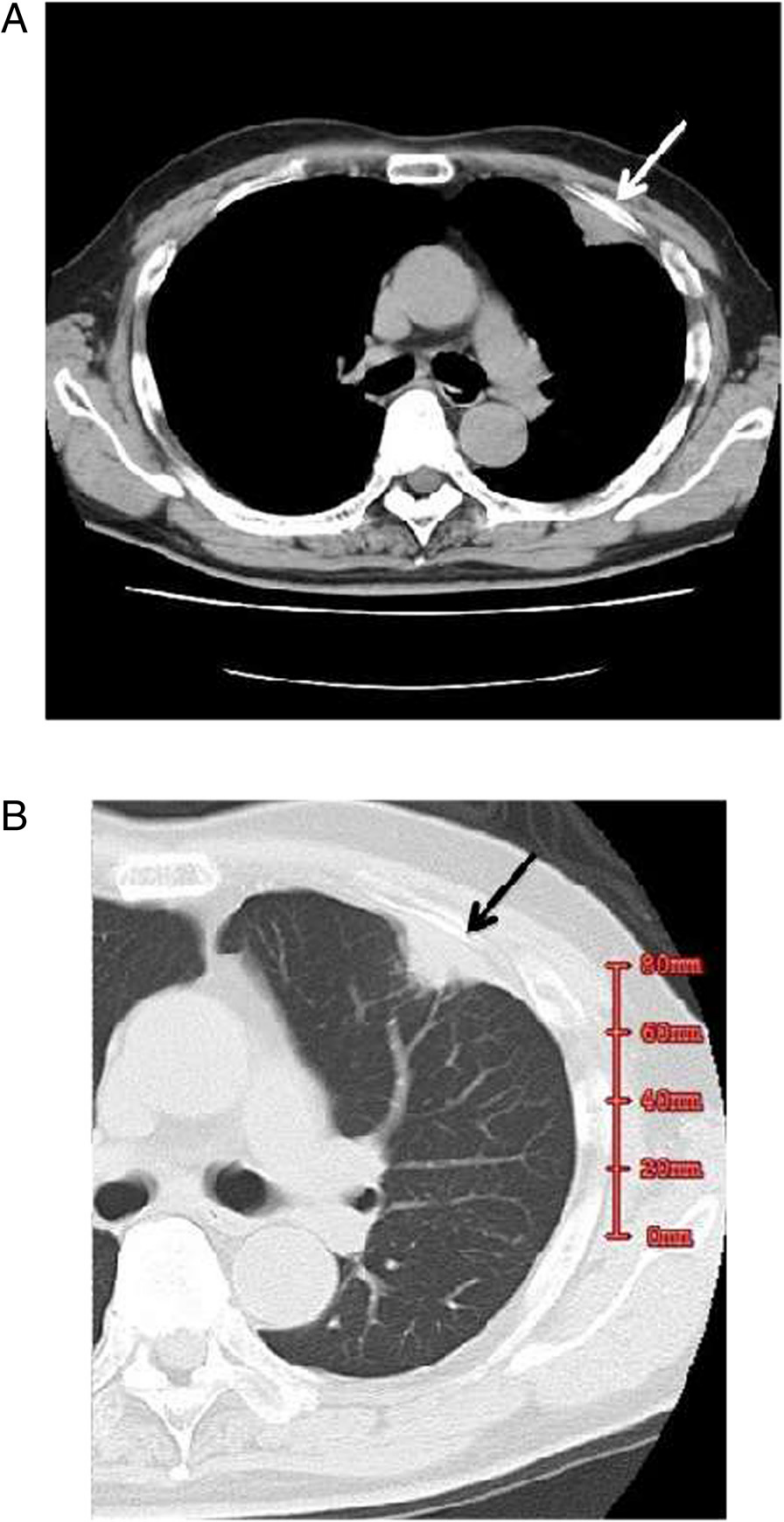
Computed tomography. **(A)** Pleural tumor is observed on the left chest wall. **(B)** Thin-slice CT shows the silhouette sign-positive tumor with an irregular surface. Arrows indicate the tumor at the pleura.

A 69-year-old man underwent a left nephrectomy for stage III 3aN0M0 RCC of clear cell type in our hospital. A total of 300 units per day of interferon-alpha (IFN-α) was administered for 1 year as postoperative chemotherapy. While the patient had not reported any specific complaints at 6 years post operation, chest CT revealed a 25 × 15 mm irregularly shaped tumor located at the anterior chest wall, directly behind the third left rib (Figure 1). Fine-needle aspiration biopsy of the tumor was performed, and histopathological examination suggested that it was an RCC metastasis. Further examination of the abdominal CT and bone scintigraphy were performed to detect additional metastases. No lesions were detected in addition to the pleural metastasis.

**Figure 2:**
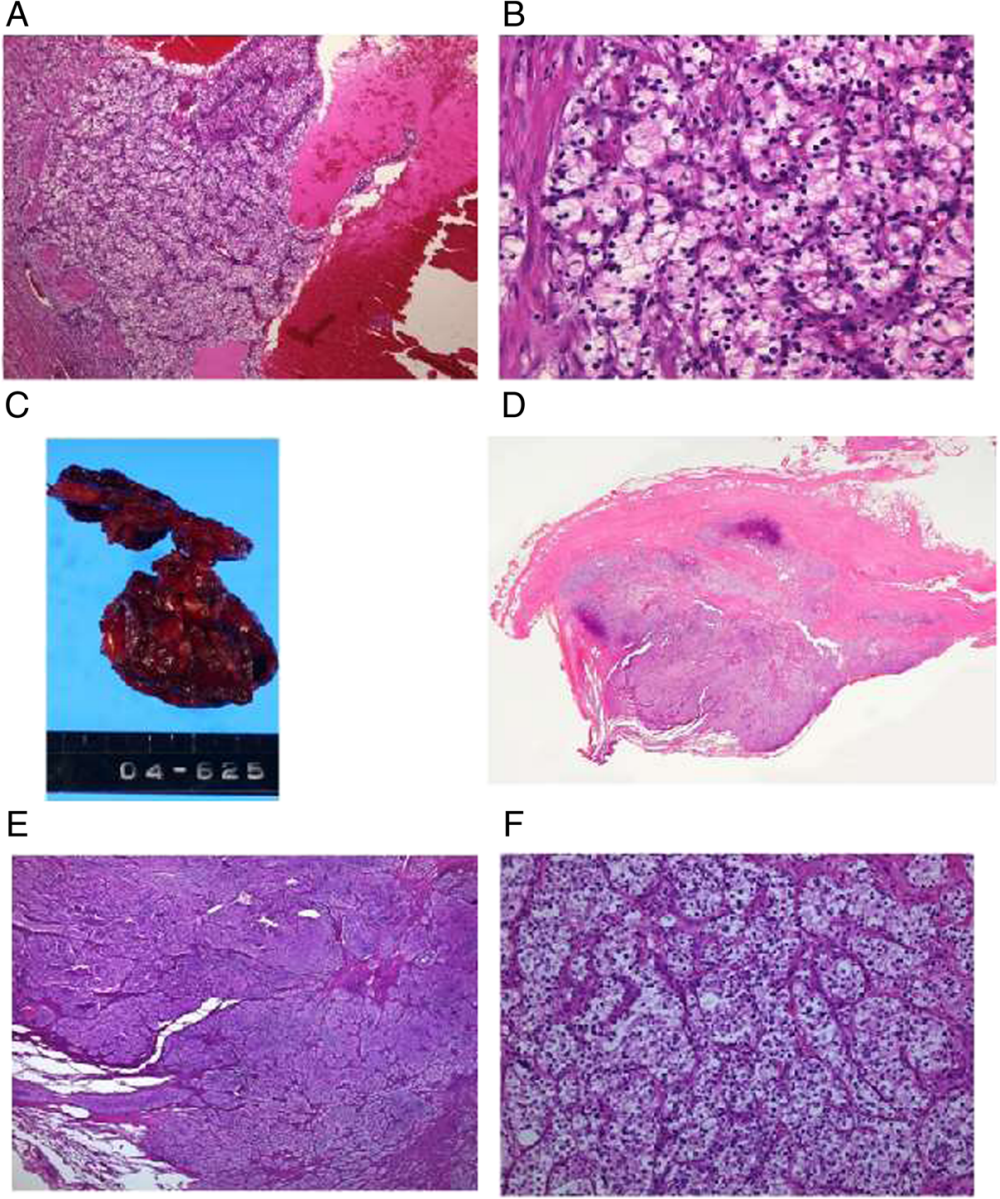
Microscopic findings of primary and metastatic pleural tumors. The lesion comprised tumor cells with abundant clear cytoplasm and small round nuclei with atypia in both primary tumor and metastatic tumor at the pleura (hematoxylin and eosin stain). **(A)** Primary lesion of the kidney (low magnification). **(B)** Primary lesion of the kidney (high magnification). **(C)** Resected tumor with rib and lung. **(D)** Histological specimen by magnifying glass. **(E)** Metastatic pleural tumor (low magnification). **(F)** Metastatic pleural tumor (high magnification).

The tumor was surgically resected in conjunction with partial resection of the third left rib and left lung. Macroscopically, the resected tumor revealed to be a 2.0 × 1.3 cm yellowish tumor. Microscopically, the lesion consisted of tumor cells growing in an alveolar pattern, separated by struma, and endowed with prominent sinusoid-like vessels. These tumor cells had abundant clear cytoplasm and uniform, small, ovoid hyperchromatic nuclei. These histological features were similar to those of the previously resected RCC lesion (Figure 2). The tumor was diagnosed as being compatible with metastatic RCC of the pleura. The postoperative course was uneventful.

IFN-α was administered for 1 year as postoperative therapy. The patient has been alive for 7 years without recurrence of the carcinoma.

## Discussion and conclusions

Metastasis to the lungs or mediastinum may already be present in more than one third of patients with RCC at the time of diagnosis [3]. Although RCC commonly metastasizes to the lung, pleural metastasis is rare. Saitoh et al. [4] have investigated 1,451 autopsy cases of RCC. Of these, 979 cases (76%) had lung metastases, and 154 cases (12%) had pleural metastases; there were no cases of solitary pleural metastases. Kutty et al. [3] reported 25 thoracic metastases of RCC, including 7 cases of pleural metastasis, but none of these 7 cases showed solitary pleural metastasis. According to those reports, RCC spread to parietal pleurae appears to be extremely rare.

Including the case reported in this study, 14 cases of solitary pleural metastasis from RCC have been reported [5–17] (Table 1). Of these, 1 case was not reported precisely, and 7 showed a large volume of pleural effusion at the time of diagnosis. Panpleuro-pneumonectomy was performed on 1 patient whose cancer had recurred after surgery. The other 7 patients received chemotherapy. Cases with pleural effusion had a poor prognosis. From a literature search in PubMed and Japanese Centra Revuo Medica databases, we found that the present case is the first reported case of solitary localized pleural metastasis of RCC without pleural effusion and long-term survival after resection.

**Table 1:** **Reported cases of solitary metastasis to the pleura from renal cell carcinoma**

**Reference**	**Age**	**Sex**	**Characteristic of lesion**	**Therapy**	**Prognosis**
Latour and Shulman [5]	Unknown	Unknown	Unknown	Unknown	Unknown
Taylor et al. [6]	69	M	Pleural tumor with huge pleural effusion	BSC	Dead at 4 months
Ishida et al. [7]	71	M	Pleural tumor with huge pleural effusion	BSC	Dead at 2 months
Yamasaki et al. [8]	61	M	Pleural tumor with huge pleural effusion	Unknown	Unknown
Nakamura et al. [9]	50	M	Pleural tumor with huge pleural effusion	Operation	Unknown
Azuma et al. [10]	74	M	Pleural tumor with pleural effusion	Unknown	Dead at 3 months
Ohnishi et al. [11]	66	M	Huge pleural effusion	IFNα	Unknown
Kamiyoshihara et al. [12]	68	M	Pleural tumor with huge pleural effusion	IFNα/RT	Dead at 15 months
Mizunuma et al. [13]	59	M	Pleural tumor with huge pleural effusion	BSC	Dead at 2 months
Kragel and Wei [14]	58	M	Metastasis to solitary fibrous tumor of the pleura	Operation	Dead at 6 months
Kang et al. [15]	51	M	Pleural tumor with pleural effusion	Unknown	Unknown
Sun et al. [16]	50	M	Multiple nodules with pleural effusion	Chemotherapy	Alive at 8 months
Yoshii et al. [17]	67	M	Pleural tumor with huge pleural effusion	Chemotherapy	Alive at 10 months
Our case	75	M	Pleural tumor	Operation	Alive at 92 months

BSC, best supportive care; RT, radiation therapy.

To date, there is no effective chemotherapy against RCC except interferon therapy; therefore, surgical resection is preferable for localized metastasis thereof. Takashi et al. [18] reported 21 cases of surgical resection of metastatic lesions from RCC. In that report, patients with complete resection of metastasized lesions showed favorable prognoses.

Since the metastatic lesion in our case was solitary and small, surgical resection was performed. Furthermore, the metastatic lesion was detected before the patient complained of any symptoms. By the time symptoms are evident to the patient, the extent of disease is likely to be very advanced (growth of lesion and pleural effusion) and thus incurable. Curative treatment in patients with malignant pleural effusion is quite difficult. Routine CT examination proved very useful in finding the pleural metastatic lesion at the localized stage in this case study. In the future, CT may also prove useful for the detection of lung metastases. Routine monitoring for potential chest metastases by CT and other methods should also be considered, in the context of ‘rescue’ of patients with a solitary detectable metastasis.

## Consent

Witten informed consent was obtained from the patient for the publication of this case report and any accompanying images. A copy of the written consent is available for review by the Editor-in-Chief of this journal.
